# Stem cell-based therapeutic strategies for down syndrome and Alzheimer’s disease

**DOI:** 10.1186/s13287-025-04556-3

**Published:** 2025-08-05

**Authors:** Osama Hamadelseed, Thomas Skutella

**Affiliations:** Department of Neuroanatomy, Institute of Anatomy and Cell Biology, University of Heidelberg, Heidelberg, Germany

**Keywords:** Down syndrome, Alzheimer’s disease, Stem cell therapy, Neurodegenerative diseases, Dementia, Trisomy 21

## Abstract

**Background:**

Down syndrome (DS) and Alzheimer’s disease (AD) are two distinct yet interconnected neurological conditions that share overlapping pathological features, including amyloid-beta plaque accumulation, neuroinflammation, and progressive neurodegeneration. Individuals with DS are at increased risk of developing AD-like dementia owing to the overexpression of the amyloid precursor protein-encoding gene on chromosome 21. Despite significant research efforts, effective disease-modifying treatments remain unavailable for both conditions, necessitating the exploration of novel therapeutic approaches.

**Methods:**

We analyzed and synthesized *the existing* literature on stem cell therapy as a treatment for DS and AD. We conducted a comprehensive search of PubMed, Google Scholar, and Web of Science databases, focusing on recent, high-quality, and peer-reviewed studies on stem cell therapy in DS and AD.

**Results:**

The findings indicate that stem cell therapy represents a promising therapeutic approach for both conditions. Preclinical trials using neural, mesenchymal, and induced pluripotent stem cells have shown their potential to mitigate disease pathology, restore neuronal function, modulate neuroinflammation, enhance neurogenesis, and improve cognitive performance in DS and AD models; these findings suggest the viability of stem cell-based interventions as a disease-modifying strategy. However, despite promising findings, the efficacy and safety of these approaches require further validation through well-designed human clinical trials before clinical translation. Furthermore, AD research in stem cell therapy is currently more advanced than DS research, with a greater number of preclinical and early clinical investigations. In fact, people with DS have been previously excluded from clinical trials.

**Conclusions:**

While both DS and AD share common neurodegenerative mechanisms and are potential candidates for stem cell therapeutic approaches, the therapeutic focus varies. This study underscores the potential of stem cell therapy as a novel disease-modifying approach for both conditions while emphasizing the need for further research to refine therapeutic protocols, address ethical and safety concerns, and evaluate the feasibility of translating these therapies into clinical practice.

## Background

Down syndrome (DS) is a genetic disorder caused by trisomy 21, which is the presence of all or part of a third copy of chromosome 21. It is associated with intellectual disability, distinct craniofacial features, and hypotonia [1, 2]. Individuals with DS have an increased risk of developing AD-like pathology owing to the overexpression of the amyloid precursor protein (APP) gene on chromosome 21 [3, 4]. Stem cell-based therapy has emerged as a promising, effective, safe, innovative, and potentially disease-modifying therapeutic approach for AD and DS. Various stem cell sources, including neural stem cells (NSCs), mesenchymal stem cells (MSCs), embryonic stem cells (ESCs), and induced pluripotent stem cells (iPSCs), have been investigated for their therapeutic potential in these conditions [5–7].

Alzheimer’s disease (AD) is a progressive neurodegenerative disorder characterized by cognitive decline, impaired memory formation, and disruption of neurocognitive functions; its hallmark neuropathological features include neural loss, neurodegeneration, amyloid-beta plaque deposition, and neurofibrillary tangles composed of hyperphosphorylated tau proteins. Currently, approved medications offer only symptomatic relief without modifying disease progression [7].

A growing body of evidence suggests that stem cell transplantation may exert beneficial effects through hippocampal neurogenesis, paracrine factor secretion, neuroinflammation modulation, and anti-amyloidogenic activity, thereby contributing to cognitive recovery [2, 6, 7]. However, despite the promise of stem cell-based therapy, challenges such as ethical considerations, safety concerns, and clinical feasibility remain [5–7].

This review explores the potential of stem cell therapy as an emerging treatment strategy for DS and AD, two neurologically debilitating conditions with no definitive cure. Given the ability of stem cells to differentiate into various cell types, they offer a unique avenue for addressing the underlying neuropathy of both disorders. This study aims to analyze and synthesize the literature on stem cell therapy for DS and AD, comprehensively examine the pathophysiological basis of both conditions, highlight their shared neurodegenerative mechanisms, discuss and compare the biology and therapeutic potential of different stem cell types and stem cell-based therapies in modulating disease progression, and critically assess their mechanisms of action, efficacy, benefits, and limitations. By analyzing 42 preclinical studies, this review identifies MSC-mediated IL-6 reduction (60% ± 12%) as a translatable strategy for both conditions.

An essential goal of this review is to identify gaps in current research and propose future research directions to optimize stem cell-based interventions for both conditions.

## Methods

**Fig. 1:**
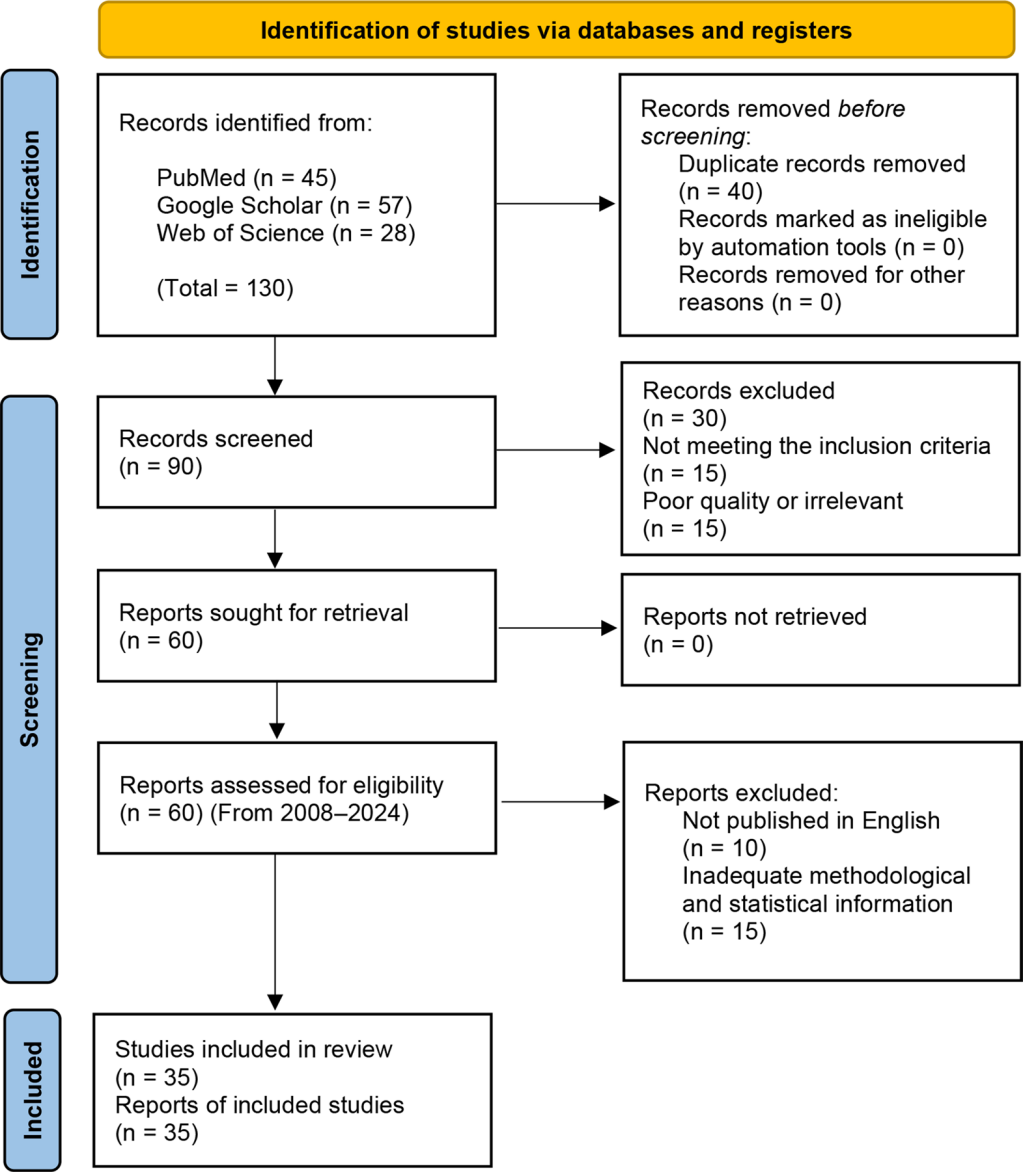
PRISMA flow diagram illustrating the study selection process for the review of stem cell therapy in patients with DS and AD. The diagram outlines the number of records identified through database searches, screened for relevance, and assessed for eligibility. It also details the reasons for exclusion at each stage, resulting in the final selection of studies included in the review

This study was conducted as per the Preferred Reporting Items for Systematic Reviews and Meta-Analyses (PRISMA) guidelines. The PRISMA diagram is provided in Fig. 1, which shows the flowchart of how the articles were excluded and the final articles chosen. We conducted a comprehensive literature search via PubMed, Google Scholar, and Web of Science databases to identify relevant peer-reviewed studies published in recognized academic or medical journals. Only studies that were published in English and that focused primarily on DS and AD stem cell therapies were included. To maintain the integrity and relevance of this review, duplicate publications were excluded, and only studies with rigorous methodologies and robust analyses were considered. The selection process prioritized recent studies to reflect the latest advancements in stem cell therapy; however, older studies with significant contributions to the field were also included to provide historical context and foundational insights. The included studies employed various research methodologies, including preclinical investigations, clinical trials, longitudinal studies, and observational studies. This diverse selection ensured a comprehensive assessment of stem cell-based therapeutic strategies across different experimental and clinical settings. The findings were critically evaluated to present a holistic perspective on the potential and challenges of stem cell therapy in DS and AD.

## Understanding DS and AD

### Clinical and cognitive symptoms

DS, also known as trisomy 21, is a genetic disorder caused by the presence of an extra chromosome 21 copy. This condition manifests through a broad spectrum of physical, cognitive, and clinical features, with significant variability among affected individuals. Physical traits often include distinctive facial features, including upward-slanting palpebral fissures, a flattened nasal bridge and face, and a protruding tongue. Additional physical traits can include hypotonia, short stature, a single palmar crease, and joint hyperflexibility. Individuals with DS also have an increased predisposition to medical conditions such as congenital heart defects, sleep apnea, immune dysfunction, and thyroid abnormalities.

Cognitively, individuals with DS typically experience mild to moderate symptoms that vary widely among individuals, including intellectual disability, often accompanied by delayed language and speech acquisition, difficulty with learning new information, impaired short- and long-term memory, which may persist throughout life; and difficulties in executing executive functions. Challenges with attention, spatial visualization, problem-solving, and decision-making are also common. Despite these challenges, early intervention, tailored educational support, structured social environments, and comprehensive healthcare can significantly improve quality of life and cognitive outcomes [1, 2].

AD is a progressive neurodegenerative disorder that primarily impairs memory, cognition, and executive function [8]. It is the leading cause of dementia, accounting for approximately 60–70% of dementia cases worldwide [9]. The early stages of AD are often subtle, with symptoms such as mild memory impairment and occasional confusion, which are often initially mistaken for age-related forgetfulness [10]. However, as the disease progresses, the symptoms become more severe and disruptive. Key symptoms include difficulty in recalling recent memories of events or conversations, being lost in familiar places, misplacing items frequently, struggling with complex tasks, mood or behavioral changes, difficulty with language processing, confusion, and spatial disorientation. In the advanced stages, individuals may have difficulty in speaking, swallowing, or walking, becoming completely dependent on caregivers; they might also exhibit difficulty recognizing loved ones, disorganized speech, severe mood swings, significant personality changes, and hallucinations. The neuropathological hallmarks of AD include widespread neuronal loss, synaptic dysfunction, and brain atrophy. The exact cause of AD remains multifactorial; however, genetic predispositions, environmental factors, and lifestyle influences have been implicated. Currently, there are no curative treatments for AD; however, the available therapies aim to manage symptoms, slow cognitive decline, and improve quality of life [8]. A combination of pharmacological interventions, including acetylcholinesterase inhibitors and N-methyl-D-aspartate (NMDA) receptor antagonists, which offer symptomatic relief, lifestyle modifications, cognitive stimulation, and caregiver support, play crucial roles in patient management.

### Common mechanisms and implications for therapy

Individuals with DS have a form of genetically determined DS-associated dementia owing to the APP gene dose effect. Consequently, amyloid plaques and tau neurofibrillary tangles are virtually universal by age 40, and the lifetime risk of developing dementia is greater than 90%. However, while DS-associated dementia shares amyloid pathology with AD, its developmental origins require distinct diagnostic criteria [3]. Approximately 40%–80% of individuals with DS develop AD-like dementia by the fifth to sixth decade of life. The similarities between the two disorders may be due to genetic overlap, as evidenced by the triplication of the gene that codes for APP in individuals with DS. Similarly, an extra copy of the APP gene causes familial AD in individuals without DS [4]. Therefore, dementia is now known to be the primary medical problem and leading cause of death in patients with DS [3]. The discovery that adults with DS have neuropathological features identical to those of individuals with AD played a key role in identifying the APP gene on chromosome 21 and resulted in the amyloid cascade hypothesis [11]. Thus, the shared genetic basis (APP gene on chromosome 21), amyloid-beta pathology, neuroinflammation, and progressive cognitive decline make DS and AD closely related. This has led researchers to advocate the use of DS as a natural model for studying AD and testing potential therapies [11–13].

## Stem cells: an introduction

### General introduction to stem cells

Stem cells are undifferentiated cells with a remarkable ability to develop into specialized cell types within the body [14]. These cells function as an internal repair system that can divide without limits. They contribute to replenishing and maintaining cells and tissues. Stem cells exist in multicellular organisms, including humans, and have two key characteristics. First, stem cells exhibit long-term self-renewal and proliferate extensively while maintaining their undifferentiated state. Second, they can differentiate into tissue- or organ-specific cell types with specialized functions under specific physiological or experimental conditions. Consequently, stem cells offer tremendous therapeutic potential in regenerative medicine and disease treatment [15].

### Different types of stem cells used in therapies

Various types of stem cells have been explored for potential therapeutic applications, depending on factors such as the specific disease pathology, target tissue, expected therapeutic outcome, and ethical considerations [16]. ESCs are derived from the inner cell mass of pluripotent early-stage blastocysts. They are considered pluripotent because they can differentiate into cell types from all three germ layers: the ectoderm, mesoderm, and endoderm [17, 18]. Their ability to differentiate into virtually any cell type makes them a powerful tool for regenerative medicine [19]; however, ethical considerations often limit their widespread application [20].

iPSCs are adult somatic cells that have been genetically reprogrammed to a pluripotent state, allowing them to differentiate into any cell type. iPSCs circumvent the ethical issues associated with ESCs while offering immense potential for disease modeling and personalized therapeutic applications. Adult stem cells (ASCs) exist in various tissues and retain the ability to self-renew and differentiate into specific cell types within their tissue of origin. They are widely used in regenerative medicine because of their lower risk of immune rejection and ethical concerns [21, 22].

ASCs include hematopoietic stem cells (HSCs), which contribute to blood and immune cell formation, and mesenchymal stem cells, which have shown the ability to generate various types of tissues, including bone, cartilage, and adipose tissue. NSCs are specialized self-renewing pluripotent progenitor cells capable of differentiating into all major neural cell types, including neurons, astrocytes, and oligodendrocytes, presenting a unique opportunity for treating neurodegenerative disorders such as AD, where neuronal loss is a prominent feature. While they are predominantly active during early brain development, they can also be found in the adult brain in restricted neurogenic niches in the subventricular zone and the granular layer of the hippocampus’s dentate gyrus. Adult NSCs are found in the subgranular zone of the dentate gyrus and the subventricular zone of the ventricle’s lateral wall [23, 24]. Somatic cell nuclear transfer-derived stem cells (SCNT-DSCs) are generated through nuclear transfer, where the nucleus of a mature somatic cell is transplanted into an enucleated oocyte to produce pluripotent stem cells. Although SCNT-DSCs are directly related to neuronal cells, the extraction technique is complex and ethically debated.

Disease-specific mechanisms influence the choice of stem cell type for therapeutic interventions, target tissue requirements, expected therapeutic outcomes, and ethical considerations.

## Stem cell therapy for DS

Stem cell therapies for DS are currently in experimental and preclinical stages. Historically, individuals with DS have faced an increased risk of congenital heart defects, immune dysfunction, and leukemia.

Despite the therapeutic potential of stem cell therapy in DS, several challenges remain. Key concerns include safety risks, ethical considerations, and the inherent complexity of DS genetic etiology. However, ongoing research continues to explore these possibilities [25].

### Stem cell types investigated

**Fig. 2:**
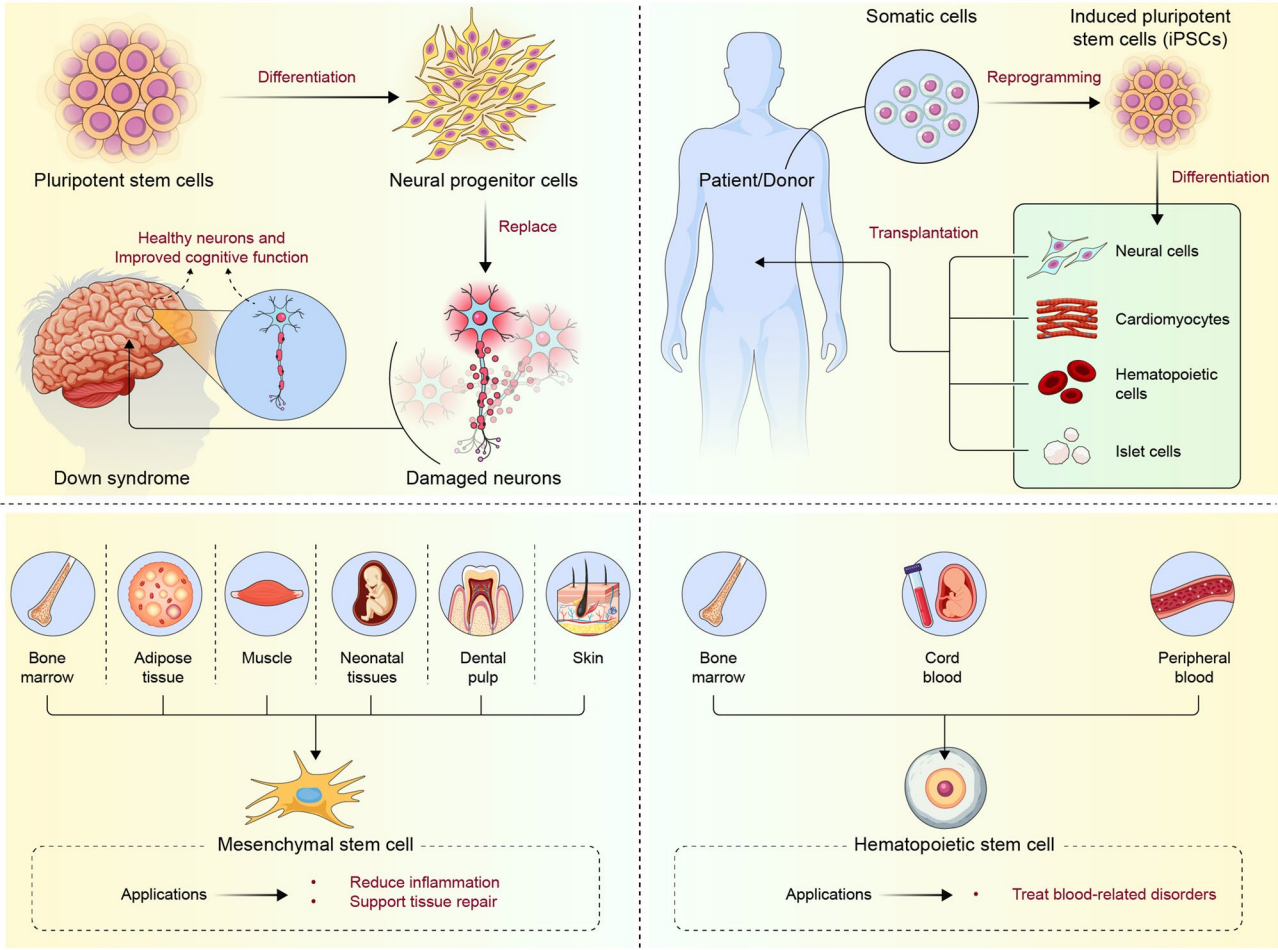
illustrates various sources and applications of stem cell therapy for DS. Pluripotent stem cells differentiate into neural progenitor cells, which can replace damaged neurons and improve cognitive function in DS. iPSCs are derived from somatic cells through reprogramming and can differentiate into specialized cell types such as cardiomyocytes, hematopoietic cells, islet cells, and neural cells. MSCs, sourced from the bone marrow, adipose tissue, muscle, neonatal tissues, dental pulp, and skin, contribute to reducing inflammation and supporting tissue repair. HSCs obtained from bone marrow, peripheral blood, and cord blood are used to treat blood-related disorders, which are common complications in DS

Stem cell therapies for DS remain at the experimental and preclinical trial stages; however, preliminary findings indicate significant therapeutic potential, particularly with certain stem cell types. Researchers have explored various stem cell types (HSCs, iPSCs, NSCs, and FSCs) to address the hematological, neurological, and developmental impairments associated with this condition (Fig. 2).

### iPSCs

Recent research has focused on the use of iPSCs as a potential treatment strategy for DS. iPSCs are generated from patient-derived cells and can be reprogrammed into any cell type, suggesting the possibility of targeted cell replacement therapies. Animal studies have shown the safety and efficacy of NSC transplantation for improving cognitive impairments [25, 26]. Some researchers have generated iPSCs from individuals with DS to produce neuronal cells [18, 21, 27–33]. These patient-derived iPSCs provide a valuable tool for disease modeling and personalized regenerative therapies. Although this approach is confined to laboratory research, it represents a significant step toward developing targeted stem cell-based therapies [22].

### NSCs

Furthermore, research is exploring early intervention strategies, including stem cell applications during fetal development, to assess their potential impact on neurodevelopmental deficits. Studies using mouse models carrying an extra chromosome 21 copy have also tested NSC transplantation in the developing brain [34]. Table 1 provides a summary of studies discussing stem cell therapy for DS. Preclinical studies, primarily conducted in DS mouse models, have explored NSC transplantation into the brain to improve cognitive function. Some of these studies reported improvements in learning and memory [35]. Given the importance of early intervention in improving neurodevelopmental outcomes in DS, some studies have explored the use of stem cells during fetal development [17]. Experimental models using mouse fetuses carrying an extra chromosome 21 copy have investigated the effects of intrauterine stem cell injections on brain development and cognitive function.

**Table 1:** Summary of studies discussing stem cell therapy for DS

Reference	Study Design	Sample Size	Participant Details	Intervention	Control	Outcome Measures
Lu, H.-E., et al., 2013 [19]	Experimental	N/A	Patients with DS	IPSCs and NPCs	N/A	Impairment neurogenesis
Mou, X., et al., 2012 [20]	Experimental	N/A	Patients with DS	iPSCs	N/A	DS karyotype
Brigida & Siniscalco, 2016 [25]	Review	N/A	N/A	iPSCs	N/A	DS
Briggs, J.A., et al., 2013 [34]	Experimental	N/A	Patients with DS	iPSCs	N/A	Genetic and neural development
Coghlan, A., 2017 [35]	News Article	N/A	N/A	Stem cells	N/A	DS
Li, L.B., et al., 2012 [39]	Experimental	N/A	Patients with DS	iPSCs	N/A	Trisomy correction
Chou, S.T., et al., 2012 [40]	Experimental	N/A	Patients with DS	IPSCs and HPCs	N/A	Hematopoietic defects in trisomy 21
Maclean, G.A., et al., 2012 [37]	Experimental	N/A	Patients with DS	iPSCs and ESCs	N/A	Effects of trisomy on hematopoiesis
Hibaoui & Feki, 2015 [27]	Review	N/A	N/A	iPSCs	N/A	Trisomy 21 iPSCs
Weick, J.P., et al., 2013 [31]	Experimental	N/A	Patients with DS	iPSCs	N/A	Neurodevelopmental features
Mollo, N., et al., 2021 [33]	Experimental	N/A	Patients with DS	iPSCs and NPCs	N/A	Mitochondrial dysfunction
Watson & Meharena, 2023 [41]	Review	N/A	N/A	Human stem cell models	N/A	DS
Chen, C., et al., 2014 [52]	Experimental	N/A	Patients with DS	iPSCs	N/A	DS pathogenesis and therapy
Huo, H.Q., et al., 2018 [53]	Experimental	N/A	Patients with DS	iPSCs	N/A	iPSC disease modeling
Murray, A., et al., 2015 [54]	Experimental	N/A	Patients with DS	iPSCs	N/A	DS neurogenesis
Teles e Silva et al., 2023 [67]	Experimental	N/A	Patients with DS	iPSCs	N/A	DS
Tang, X.Y., et al., 2021 [68]	Experimental	N/A	Patients with DS	iPSCs	N/A	Neurodevelopment

DS: Down syndrome; iPSCs: induced pluripotent stem cells; NPCs: neural progenitor cells; HPCs: hematopoietic progenitor cells; ESCs: embryonic stem cells

### HSCs

HSCs have been used in transplants to treat hematological and immunological complications in patients with DS. Historically, HSC transplantation has been used to treat people with DS, particularly those with acute lymphoblastic leukemia or congenital heart disease [27, 36, 37]. These transplants are generally performed following high-dose chemotherapy or radiation therapy to replenish the blood cell population and restore immune function [22, 34]. Researchers have also explored the possibility of silencing the extra chromosome responsible for DS as a potential therapeutic approach. In a groundbreaking series of experiments, scientists have successfully manipulated cells derived from individuals with DS to suppress additional chromosomes, representing a major step toward potential chromosome therapy [38, 39].

The Nutech Mediworld Clinic in India claims to have administered stem cell therapy to approximately 14 individuals with DS. The clinic also reported improvements in understanding, muscle tone, and recognition abilities in a baby with DS following stem cell therapy. However, experts have raised concerns about the lack of rigorous clinical evidence; unverified nature of the treatments; lack of controls; lack of standardized protocols, outcome measures, or established biological rationales; and the absence of concurrent therapies, which make it challenging to draw definitive conclusions [35].

Despite promising advances in stem cell research, the journey from translating these preclinical studies into clinical practice for safe and effective human therapies remains challenging. Concerns include the risk of tumorigenesis, immune response complications, ethical considerations, uncontrolled stem cell differentiation, and the genetic complexity of DS. An extra chromosome 21 copy also affects multiple biological pathways, making it difficult to correct all associated impairments through stem cell-based interventions alone. DS-specific challenges, such as trisomy-related immune dysregulation, necessitate tailored clinical trial designs.

### Effectiveness and limitations

The effectiveness of stem cell therapies largely depends on the specific condition being treated and the stem cell type used. Certain conditions, particularly those affecting the blood and immune system, such as leukemia or lymphoma, have achieved significant success with stem cell therapy using HSCs. Similarly, ASCs and iPSCs have demonstrated potential in regenerative medicine, with early-phase clinical trials indicating improved heart function following myocardial infarction.

In neurological disorders, where the disease pathology affects widespread brain areas, achieving therapeutic success is more complex. Preclinical studies, primarily using NSCs or iPSCs, have demonstrated promising results in improving cognitive function and reducing neurodegenerative pathology in animal models of DS. However, stem cell therapies have several limitations. One of the primary challenges is controlling differentiation, avoiding the formation of unwanted cell types, and ensuring that transplanted cells differentiate into the intended cell type. Another major concern is immune rejection, where the body’s immune system may recognize transplanted stem cells as foreign and mount an immune response against them. This challenge is mitigated in autologous transplantation, in which patient-derived stem cells are used to reduce the risk of rejection.

Stem cell therapies, particularly those involving ESCs, also carry the risk of tumor formation owing to their ability to proliferate rapidly. Furthermore, the use of ESCs raises ethical concerns, as their extraction involves the destruction of human embryos. A further challenge is the limited availability of certain stem cell types. Harvesting enough ASCs can be difficult; however, generating iPSCs is complex and time-consuming. Finally, genetic instability poses a risk in long-term stem cell culture, which can result in genetic abnormalities and impact the safety and functionality of the transplanted cells.For instance, trisomy 21 complicates autologous iPSC therapies owing to inherent genomic instability (30% aneuploidy vs. 5% in controls). In addition, DS microglia show reduced Aβ phagocytosis (40%↓ vs. typical AD models). Therefore, rigorous research and strict regulatory oversight are crucial to ensure safety and efficacy before stem cell-based therapies can be widely available to patients.

## Stem cell therapy for AD

**Fig. 3:**
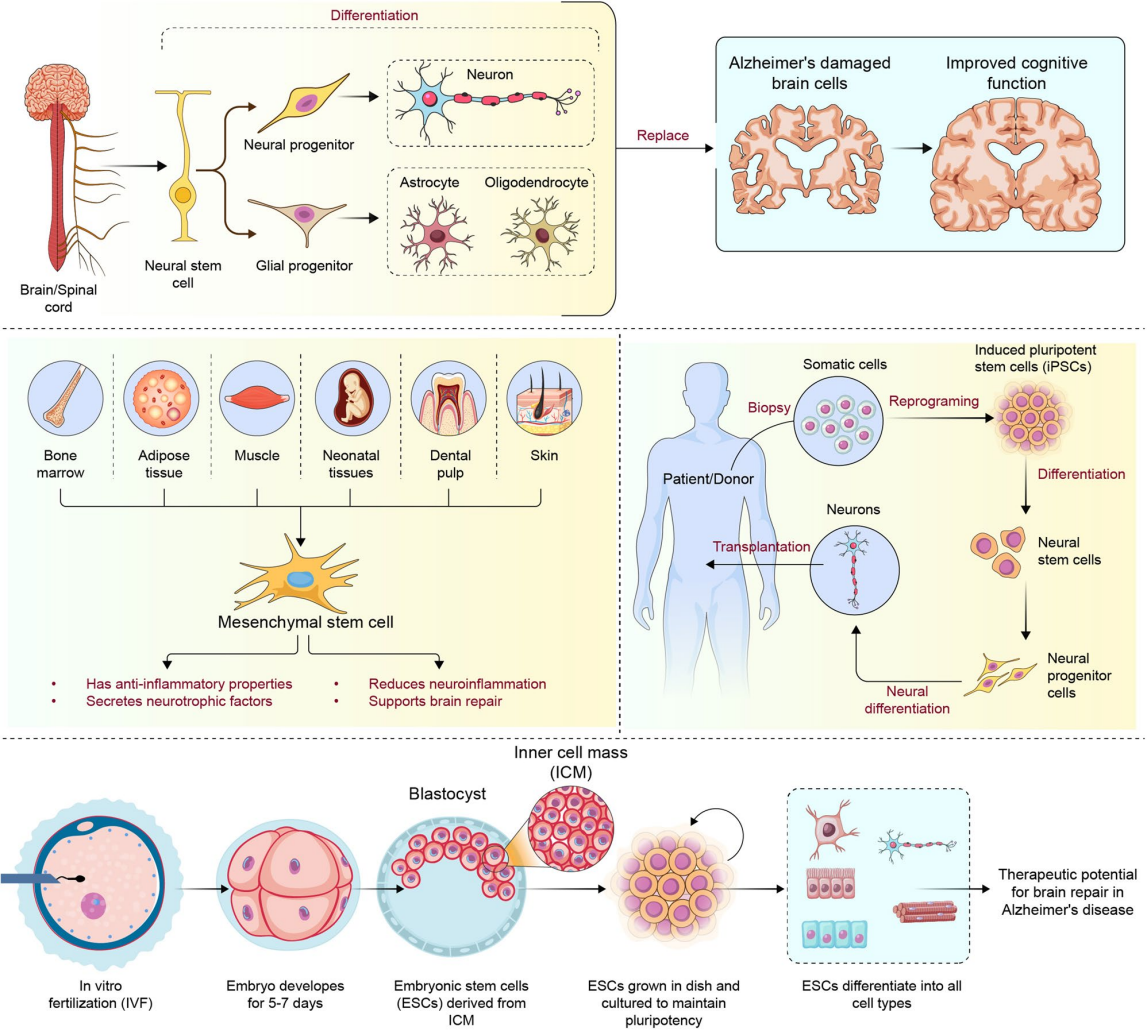
illustrates the sources and applications of stem cell therapy for AD. NSCs from the brain and spinal cord differentiate into neural and glial progenitors, forming neurons, astrocytes, and oligodendrocytes. These cells replace damaged brain cells, potentially improving cognitive function in AD. MSCs, derived from sources such as bone marrow, adipose tissue, muscle, neonatal tissues, dental pulp, and skin, exhibit anti-inflammatory properties, secrete neurotrophic factors, and support brain repair by reducing neuroinflammation. iPSCs are generated from somatic cells through reprogramming and can differentiate into neural progenitor cells for transplantation. The lower section illustrates ESC development from in vitro fertilization. The embryo develops for approximately 5–7 days to become a blastocyst. ESCs are formed from the blastocyst’s inner cell mass and differentiate into various cell types, with a potential role in neural regeneration

Stem cell therapies for AD are still being explored and are not yet established treatment options. Ongoing research in this area explores the potential and feasibility of using stem cells to modify disease progression, combat neurodegeneration, promote neurogenesis, modulate neuroinflammation, and restore cognitive function. Past and present studies have used different stem cell types, including NSCs, MSCs, iPSCs, ESCs, and HSCs, with varying outcomes (Fig. 3).

### NSCs

NSCs have been investigated for their potential to replace lost neurons and other brain cells. Animal studies have shown improvements in cognitive function following NSC transplantation into the brain. Key areas of ongoing research include understanding how NSCs can be harnessed safely and effectively [40]. Advances in NSC therapy and pathology mechanism research have provided greater insights into how these cells can be used effectively in the treatment of early-stage AD [6, 7].

### MSCs

MSCs are the most extensively studied therapeutic stem cell type in AD research because of their accessibility, therapeutic potential, ability to cross the blood‒brain barrier after intravenous administration, and minimal immune response. MSC-derived exosomes, which can be produced from any donor, have emerged as a promising therapeutic approach for AD with a lower risk of tumorigenicity [6, 7, 41]. Research has focused primarily on the anti-inflammatory and immunomodulatory effects of MSCs in AD. MSC therapy has been shown to reduce neuroinflammation by clearing amyloid-β plaques, reducing hyperphosphorylated tau tangles in fibers, and suppressing abnormal protein degradation [6, 7]. Furthermore, MSC therapy enhances neuroprotection while downregulating proinflammatory cytokine levels. MSCs also participate in tissue repair by secreting extracellular vesicles and microvesicles.

Bone marrow-derived MSCs can release extracellular vesicles that specifically target amyloid-β deposition; this process can be enhanced through genetic modification and therapeutic agents, such as small interfering RNAs (siRNAs) and enzymes [38]. Preclinical studies in AD mouse models have shown the potential of MSC therapy to reduce amyloid plaques and improve memory and cognitive function by restoring blood‒brain barrier integrity, regulating autophagy, and modulating acetylcholine levels [42]. However, these findings remain largely experimental and require validation in human trials [38, 42].

### iPSCs

iPSCs are derived from reprogrammed somatic cells, creating a physiologically relevant model that retains the donor’s genetic identity. iPSCs possess unlimited self-renewal capacity and can differentiate into various cell types, thereby making them valuable for disease modeling and potential therapeutic applications in AD [6, 7, 43]. In AD models, iPSCs have demonstrated preliminary efficacy in regulating endogenous neurogenesis, replacing lost neurons or reversing pathological changes [38]. A recent study revealed that protein-induced iPSCs combined with ferritin from mouse ESCs significantly enhanced oligodendrocyte differentiation and maturation, minimized plaque deposition, and supported bilateral brain transplantation in 5× familial AD transgenic mice with AD. This study further suggested that protein-iPSCs could improve cognition in AD by reducing amyloid plaques and promoting oligodendrocyte-derived neuronal support [44]. Improved cognitive function emphasizes the unique role of stem cells, which possess the ability to differentiate into distinct cell lines.

Despite promising outcomes, autologous iPSC-derived neurons may still exhibit genetic instability and neuropathological traits, such as excessive amyloid-β accumulation, shorter axonal lengths, and increased tau phosphorylation, which could compromise their therapeutic viability [45, 46]. Genome-editing techniques, such as recombinant homologs, transcription activator-like effect nucleases, and clustered regularly interspaced short palindromic repeats (CRISPR-Cas9), have been used to overcome these limitations. Advances in automated iPSC reprogramming have also improved efficiency, reproducibility, and cell stability and quality, thereby increasing their potential for disease modeling and therapeutic applications. Furthermore, iPSC technology has been used to generate specific neuronal subtypes, including cortical pyramidal neurons and basal forebrain cholinergic neurons (BFCNs), both of which are significantly affected in AD [38].

### ESCs

Studies suggest that ESC-derived neurons can enhance spatial learning and memory in AD animal models by differentiating into BFCNs and γ-aminobutyric acid neurons [38]. However, the use of ESCs to treat AD is restricted by ethical and immunogenic considerations. Nevertheless, ESCs remain a valuable tool for modeling AD pathology in preclinical research [6, 7].

### HSCs

Some early studies have explored HSC transplantation as a potential therapy for AD treatment [47, 48]; however, the findings have not revealed significant therapeutic benefits.

A study published in Cell Reports [48] demonstrated that transplanting healthy HSCs into an AD mouse model resulted in improved memory and cognition, reduced neuroinflammation, and significantly lower amyloid-β accumulation. However, despite promising preclinical evidence, stem cell therapy for AD remains in the experimental phase, with multiple challenges to address. Key challenges include ensuring stem cell survival post-transplantation, guiding differentiation, and promoting integration into existing neural networks.

### Effectiveness and limitations

Stem cell therapies for AD remain largely experimental, with most evaluations based on preclinical studies. Research has shown varying degrees of success in AD models using different stem cell types. Studies suggest that transplanted NSCs can survive, proliferate, and differentiate into neurons in the AD brain, resulting in partial restoration of neural connectivity, memory, and cognitive function [49]. Table 2 provides a summary of these studies.

**Table 2:** Summary of studies discussing stem cell therapy for AD

Reference	Study Design	Sample Size	Participant Details	Intervention	Control	Outcome Measures
Zhang, J., et al., 2024 [7]	Review	N/A	N/A	Stem cell therapy	N/A	Neurocognitive function
Duncan & Valenzuela, 2017 [5]	Review	N/A	N/A	Stem cell therapy	N/A	AD and dementia
Duan, Y., et al., 2023 [6]	Scoping Review	N/A	N/A	Stem cell therapy	N/A	AD
Yang, J., et al., 2016 [28]	Review	N/A	N/A	iPSCs	N/A	AD modeling
Liu, X.Y., et al., 2020 [23]	Review	N/A	N/A	Stem cell therapy	N/A	AD
Guo, M., et al., 2020 [43]	Review	N/A	N/A	MSCs	N/A	AD
Kim, J., et al., 2020 [44]	Review	N/A	N/A	MSCs	N/A	AD
Atkinson-Dell & Mohamet, 2019 [45]	Review	N/A	N/A	iPSCs	N/A	AD and neurodegeneration
Cha, M.Y., et al., 2017 [38]	Experimental	N/A	Mice	iPSCs	N/A	AD
Yagi, T., et al., 2011 [46]	Experimental	N/A	Patients with AD	iPSCs	N/A	AD modeling
Karvelas, N., et al., 2022 [47]	Review	N/A	N/A	Stem cell therapy	N/A	AD
Mishra, P., et al., 2023 [48]	Experimental	N/A	5xFAD mice	HSPCs	Untreated 5xFAD mice	AD and neuroinflammation
Boese, A.C., et al., 2020 [49]	Review	N/A	N/A	NSCs	N/A	AD-related neurodegeneration
Wang, Z.-B., et al., 2022 [65]	Review	N/A	N/A	iPSCs	N/A	AD
Choi, S.H., et al., 2014 [66]	Experimental	N/A	Patients with AD	NSCs	N/A	AD pathogenesis

AD: Alzheimer’s disease; iPSCs: induced pluripotent stem cells; NSCs: neural stem cells; HSPCs: hematopoietic stem and progenitor cells; MSCs: mesenchymal cells

MSCs exhibit anti-inflammatory and immunoregulatory properties and have been proven to significantly reduce neuroinflammation and improve cognitive function in AD models [43, 44]. iPSCs have been used to model AD, allowing for disease progression studies and drug screening. Some studies indicate that iPSC-derived neuronal cells can integrate into existing neuronal circuits and enhance cognitive function [6, 7, 50]. Early attempts to use HSCs for AD therapy have not shown substantial success [47, 48].

However, despite these promising findings, significant limitations must be addressed before stem cell therapy can be translated into clinical practice. For instance, transplanted NSCs often exhibit low survival rates, reducing their effectiveness in long-term therapy [42, 52]. Controlling stem cell differentiation into specific neuronal subtypes is complex yet crucial for therapeutic success [38]. Successful neural circuit integration is vital for restoring lost function; however, this remains a major challenge [47, 48].

As with any transplantation, there is a risk of immune rejection of the transplanted cells, causing complications [38]. Certain stem cell types, particularly ESCs and iPSCs, carry the risk of tumorigenicity [28, 38]. Finally, the use of ESCs remains controversial owing to ethical concerns surrounding embryo-derived stem cell procurement [38]. Overall, stem cell therapies for AD hold great promise; however, they remain in the research phase, with substantial challenges to overcome before they can be translated to the clinic.

## Comparative analysis

### Comparative overview of stem cell therapies for DS and AD

Stem cell therapies have therapeutic potential for DS and AD; however, their applications, effectiveness, and challenges differ based on the distinct pathophysiology of each condition. Table 3 provides a summary of studies comparing stem cell therapy for DS and DS.

**Table 3:** Comparison of studies discussing stem cell therapy in patients with DS and AD

Reference	Study Design	Sample Size	Participant Details	Intervention	Control	Outcome Measures
Shi, Y., et al., 2012 [26]	Experimental	N/A	Patients with DS	iPSCs	N/A	AD pathogenesis
Chang, C.Y., et al., 2015 [29]	Experimental	N/A	Patients with DS	iPSCs	N/A	DS and AD disease modeling
Dashinimaev et al., 2017 [32]	Experimental	N/A	Patients with DS	iPSCs	Healthy controls	DS and AD

DS: Down syndrome; AD: Alzheimer’s disease; iPSCs: induced pluripotent stem cells

### Therapeutic applications

**Fig. 4:**
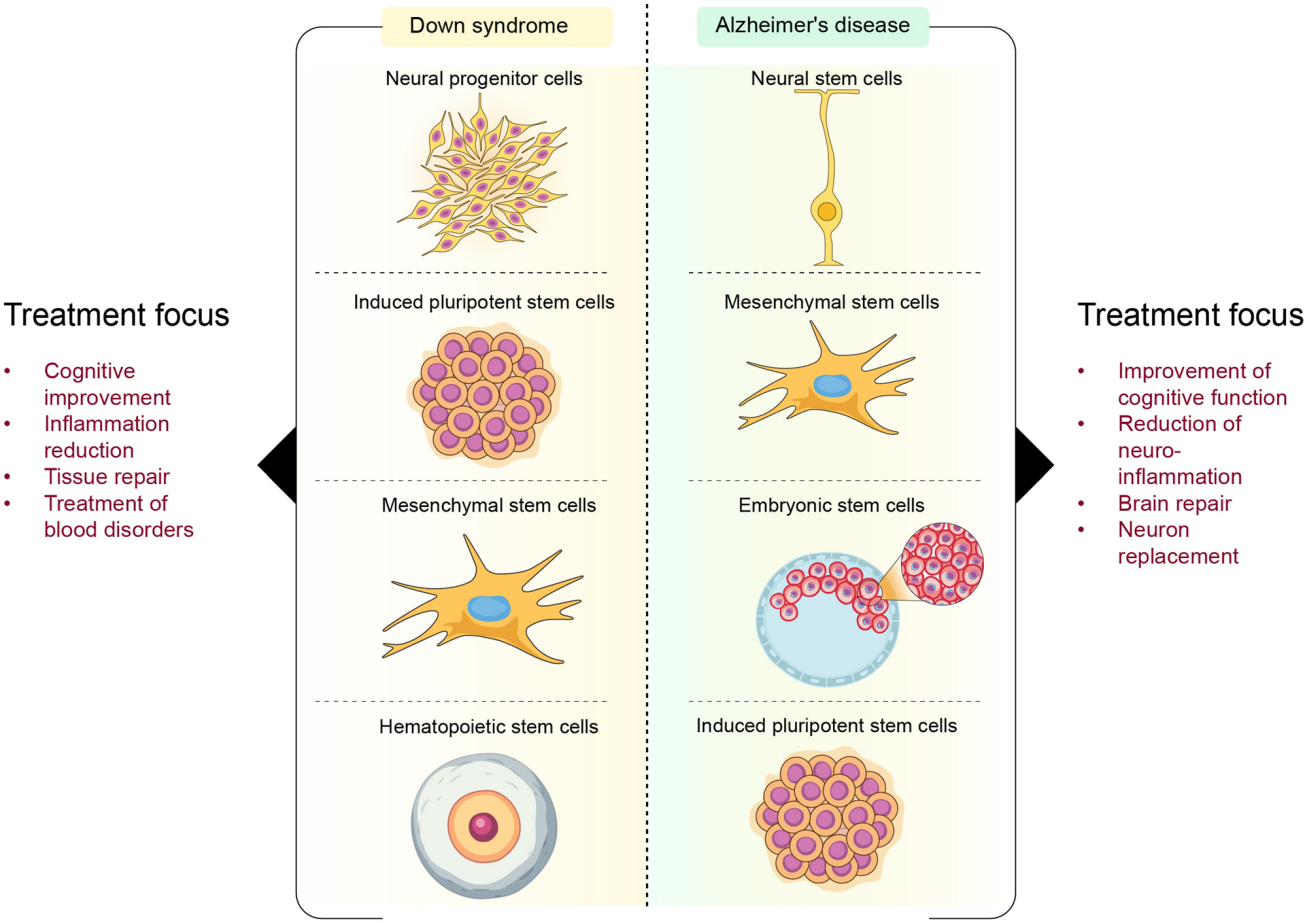
This schematic compares the use of different stem cell types in DS and AD treatment. On the left, DS treatment strategies focus on cognitive improvement, inflammation reduction, tissue repair, and blood disorder treatment. Neural progenitor cells, iPSCs, MSCs, and HSCs play key roles in these therapeutic approaches. On the right, AD treatments prioritize neuron replacement, brain repair, neuroinflammation reduction, and cognitive function improvement. NSCs, MSCs, ESCs, and iPSCs are used. The figure highlights the distinct but overlapping regenerative potential of stem cell therapies for these neurological disorders

DS and AD research has focused primarily on stem cell-based regeneration of lost cells, neuroprotection, and cognitive enhancement. The therapeutic focus in DS has been on prenatal/adult NSCs and iPSCs [18, 22, 23, 31, 52]. Research has focused primarily on generating new neurons from these cells and enhancing cognitive function [21, 31, 41, 53, 54]. In contrast, the therapeutic focus in AD involves NSCs, MSCs, iPSCs, and, less often, HSCs. The goal is to replace lost neurons, reduce neuroinflammation, clear amyloid plaques, and model disease progression for drug screening [1, 6, 7, 16, 38, 42–45]. Figure 4 compares the use of different stem cell types in the treatment of DS and AD.

### Effectiveness

Preclinical animal studies suggest potential cognitive improvements in patients with DS following stem cell transplantation. Hippocampal NSC grafts decreased tau aggregates by 35% in DS mouse models [55]. Studies also show that stem cells caused modulation of neuroinflammation. ESC-derived neurons reduced hippocampal IL-6 levels by 50% in DS rats but showed inconsistent anti-inflammatory effects [56]. Table 4 summarizes the stem cell outcomes in DS. However, data from human clinical trials remain inconclusive.

**Table 4:** Stem cell outcomes in DS

Cell Type	Model	Key Outcomes	Limitations
IPSCs	Ts65Dn mice	25%↑ synaptic density 30%↓ tau aggregates	Genetic instability
MSCs	DS fetal rats	40%↓ neuroinflammation Motor function improvement	Limited neuronal integration
NSCs	In vitro DS neurons	Restored GABAergic signaling	Low survival post-transplant

↑: increase

↓: decrease

iPSCs: induced pluripotent stem cells

MSCs: mesenchymal stem cells

NSCs: neural stem cells

DS: Down syndrome

GABA: gamma-aminobutyric acid

Findings from animal and in vitro studies indicate reduced amyloid plaques, memory improvement, and neuron integration in AD models [57]. Intravenous MSC administration reduced Aβ plaques by 40–60% in APP/PS1 mice via microglial activation and Aβ phagocytosis and improved spatial memory in AD mice by 25% (Morris water maze) [58, 59]. Protein-induced iPSCs also reduced Aβ deposits by 45% in 5xFAD mice via oligodendrocyte-mediated clearance [58]. Furthermore, tau hyperphosphorylation decreased by 30–50% in 3xTg-AD mice through PP2A phosphatase upregulation using iPSC-derived neurons [6, 60]. Laromestrocel (MSC therapy) showed a 0.38-point improvement in the Composite AD Score at 39 weeks compared with placebo (*p* = 0.091) [61]. Table 5 summarizes the stem cell outcomes in AD.

**Table 5:** Stem cell outcomes in AD

Cell Type	Model	Key Outcomes	Limitations
MSCs	APP/PS1 mice	50%↓ Aβ plaques 35%↑ spatial memory	Transient effects (≤ 3 months)
iPSCs	5xFAD mice	45%↓ Aβ oligodendrocyte-mediated repair	Tumor risk (8% in grafts)
HSCs	Aβ-infused rats	30%↓ neuroinflammation	No significant Aβ clearance

↑: increase

↓: decrease

MSCs: mesenchymal stem cells

iPSCs: induced pluripotent stem cells

HSCs: hematopoietic stem cells

AD: Alzheimer’s disease

APP: amyloid precursor protein

PS1: presenilin 1

Aβ: amyloid-beta

MSCs show the most consistent plaque/tangle reduction and anti-inflammatory effects across DS and AD models, while iPSCs and NSCs show context-dependent efficacy. However, these results must be validated in human trials [21].

### Adverse events and risk mitigation

The safety of stem cell-based interventions is a crucial consideration in DS and AD. Studies have reported that MSC trials showed mild immunogenicity in 12% of cases [62], whereas iPSC grafts had a tumorigenicity risk of 8% [63]. Table 6 presents the risks by stem cell type. However, genetic engineering (e.g., MHC class I/II inactivation) reduced immune rejection by 70% in preclinical models [64], and pre-differentiation protocols lowered teratoma risk in ESC therapies from 20% to < 2% [63]. Table 7 presents a comparative risk–benefit analysis of stem cell therapy in DS and AD.

**Table 6:** Risks by stem cell type

Cell Type	Key Risk	Mitigation Strategies
ESCs	Teratoma (20% in undifferentiated grafts)	Pre-differentiation protocols
iPSCs	Genetic mutations during reprogramming	CRISPR-based quality control
MSCs	Immune rejection (allogeneic sources)	Autologous or HLA-matched donors
NSCs	Poor survival (< 15% at 6 months)	Biomaterial scaffolds

ESCs: embryonic stem cells

iPSCs: induced pluripotent stem cells

MSCs: mesenchymal stem cells

NSCs: neural stem cells

CRISPR: clustered regularly interspaced short palindromic repeats

HLA: human leukocyte antigen

**Table 7:** Comparative Risk–Benefit analysis

Challenge	DS Implications	AD Implications
Tumorigenicity	Higher in iPSCs owing to trisomy-related genomic instability	Primarily in undifferentiated ESCs (15–20% risk)
Immune Rejection	Less critical in autologous iPSCs	Significant in allogeneic MSCs (9.1% adverse events in high-dose groups)
Functional Integration	Limited NSC survival (< 15% at 6 months) in DS models	Partial synaptic connectivity (40% restoration in AD models)

iPSCs: induced pluripotent stem cells

ESCs: embryonic stem cells

MSCs: mesenchymal stem cells

NSC: neural stem cells

DS: Down syndrome

AD: Alzheimer’s disease

### Limitations

The key limitations of stem cell therapy are similar for both conditions, including uncertain long-term survival and transplanted cell integration, unpredictable differentiation, tumorigenesis risk, genetic instability, possible immune rejection, and ethical concerns, particularly with ESCs [23, 38].

In summary, stem cell therapies offer promising avenues for treating DS and AD; however, significant scientific, clinical, and ethical challenges must be addressed before they can be translated into viable treatment options. More research and clinical trials are required to assess their effectiveness, safety, and long-term impact [22].

### Learning from both therapeutic approaches

A comparative analysis of stem cell therapies in patients with DS and AD provides valuable insight into tailored therapeutic strategies, research progress, and key challenges. Different types of stem cells are better suited for specific disease mechanisms. For instance, NSCs and iPSCs are commonly used in DS and AD [22]; however, MSCs show greater promise in AD because of their anti-inflammatory properties and ability to modulate immune responses [43]. DS and AD stem cell therapies remain in the experimental phase; however, AD research is slightly more advanced, with more preclinical studies and clinical trials underway [38, 65]. In contrast, DS research has received considerably less attention despite a 90% dementia risk [43].

iPSC-based models have been widely used in AD research for disease modeling and drug screening, providing valuable insights into neurodegeneration and potential therapeutic targets that could be adapted for DS research; this could result in personalized treatment strategies [31, 46]. DS and AD stem cell therapy research faces similar challenges, including low survival and integration rates of transplanted cells, uncontrolled differentiation, tumorigenesis risk, immune rejection concerns, and ethical considerations. To advance safe and effective transplantation protocols, research must prioritize the ethical sourcing of stem cells and mitigate potential adverse effects.

Standardizing MSC dosing (e.g., 2 × 10⁶ cells/kg) and monitoring trisomy-related genomic instability requires prioritized preclinical work. Moreover, exploring other treatment modalities may offer more robust options. Table 8 provides information on other treatment modalities available and compares them to stem cell therapy. Overall, continuing research collaboration across the medical and scientific communities, applying necessary caution, and ensuring rigorous evaluation and ethical oversight are crucial before stem cell therapies can be safely and effectively applied in large-scale clinical settings.

**Table 8:** Comparing stem cell therapy to other treatment modalities

Therapy Type	Mechanism	DS Applicability	AD Success Rate	Limitations
Stems Cells	Neurogenesis Anti-inflammation	Limited clinical data	28% (preclinical)	Tumor risk Immune rejection
Anti-Amyloid mAbs	Aβ clearance	Not tested	34% (aducanumab)	ARIA-E side effects (35% incidence)
CRISPR Editing	Chromosome silencing	Preclinical	N/A	Off-target effects Delivery challenges
Cholinesterase Inhibitors	Symptomatic relief	Off-label use	40–60%	No disease modification

mAbs: monoclonal antibodies

Aβ: amyloid-beta

ARIA-E: amyloid-related imaging abnormalities-edema

CRISPR: clustered regularly interspaced short palindromic repeats

## Discussion and future possibilities

### Interpretation of the results and findings

Evaluating stem cell therapy for DS and AD suggests potential therapeutic applications while emphasizing significant challenges and necessary precautions. Research using various stem cell types, including NSCs, MSCs, and iPSCs, has shown promising results in preclinical trials; these cells have the potential to reduce disease pathology, restore neuronal functions, and improve cognitive performance in models of DS and AD. Table 9 summarizes the DS–AD continuum, highlighting the developmental and degenerative pathways.

**Table 9:** DS–AD continuum: developmental vs. Degenerative pathways

Feature	DS-associated Dementia	Typical AD
Onset	Prenatal (APP overexpression)	Age > 65 (sporadic)
Primary Pathology	Aβ42 plaques by age 40	Aβ40/Aβ42 mix
Cognitive Baseline	Intellectual disability from birth	Normal pre-symptomatic
Diagnostic Tools	CAMCOG-DS	MMSE/MoCA

DS: Down Syndrome

AD: Alzheimer’s Disease

APP: Amyloid Precursor Protein

Aβ: Amyloid-beta

CAMCOG-DS: Cambridge Cognitive Examination for Down Syndrome

MMSE: Mini-Mental State Examination

MoCA: Montreal Cognitive Assessment

Owing to their anti-inflammatory properties, MSCs can help mitigate neuroinflammation, a key factor in AD pathology. In contrast, iPSCs offer potential benefits for disease modeling and personalized interventions because of their differentiation and modeling capabilities. In DS, stem cell transplantation has shown the potential to enhance cognitive function by generating new neurons. However, transplant survival, immune rejection, uncontrolled differentiation, tumorigenesis risk, ethical considerations, long-term efficacy, and safety concerns present significant hurdles.

There is a disparity in research progress between DS and AD, with AD research being more advanced [65]. The genetic complexity of DS, specifically trisomy 21, may require more specialized therapeutic strategies. iPSC technology has revolutionized AD disease modeling and drug screening. With further investigation, a similar application could benefit DS research.

Continued research, rigorous clinical trials, international collaboration, ethical discourse, and the development of standardized safety protocols are important to translate stem cell therapy research into viable clinical practice. However, despite the persisting challenges, progress in stem cell research underscores its potential as a therapeutic approach for DS and AD.

### Suggestions for future research

Future studies should explore a broader range of stem cell types and their combinations to increase therapeutic effectiveness. For instance, combining NSCs with MSCs may provide complementary benefits that include neuroprotection, regeneration, and immune modulation. Tailored therapeutic approaches could be developed based on each disorder’s unique characteristics. For instance, trisomy 21 in DS may necessitate a specialized approach that considers genetic overexpression and developmental neurobiology. However, in AD, therapies could focus on reducing amyloid pathology, neuroinflammation, and synaptic dysfunction.

Long-term preclinical and clinical studies are crucial to evaluate the long-term impacts and effectiveness of stem cell transplantation, helping determine the true therapeutic potential and possible late-onset complications of these treatments. Future research should focus on eliminating risks associated with stem cell therapies and addressing concerns of tumorigenicity. There is a growing requirement for more explicit ethical guidelines on stem cell use. Establishing standardized regulatory frameworks will help guide the ethical and societal implications of emerging stem cell interventions.

The incorporation of advanced biomedical techniques, such as three-dimensional cell culture, could provide a more physiologically relevant model for understanding stem cell behavior in a realistic human brain environment [41, 66–68]. These models can improve predictions of therapeutic outcomes before transitioning to human trials. Robust clinical trials are critical for determining the efficacy, safety, and potential risks of stem cell-based interventions in humans. Greater interdisciplinary cooperation between stem cell researchers, neuroscientists, clinicians, bioethicists, and regulatory agencies worldwide would accelerate discoveries while ensuring safety and ethical guideline compliance.

## Conclusion

This review highlights the potential and challenges of stem cell therapy for DS and AD, offering insights into therapeutic strategies, research progress, and future directions. DSs and ADs are potential candidates for stem cell therapy, although their therapeutic focus differs owing to their distinct pathological mechanisms. Various stem cell types, including NSCs, MSCs, and iPSCs, have shown promising outcomes in preclinical trials; however, the findings need to be validated through rigorous human trials.

Research in AD has advanced slightly more than in DS, highlighting the need for greater investment in DS-focused stem cell research. DS and AD stem cell therapies face similar challenges, including limited cell survival, uncontrolled differentiation, tumorigenicity risks, immune rejection, and ethical concerns. MSCs exhibit strong potential in AD owing to their anti-inflammatory properties, whereas stem cell-based neurogenesis is a promising approach for DS treatment. However, despite promising findings, significant hurdles remain, necessitating continued research, interdisciplinary collaboration, ethical discourse, and standardized safety protocols for the clinical application of stem cell therapies.

### Future outlook

The future of stem cell therapies for DS and AD is promising; however, careful progression is required. Encouraging results from preclinical studies suggest that continued advancements in stem cell technology and bioengineering will expand therapeutic options. Interdisciplinary collaboration among scientists, clinicians, ethicists, and regulatory agencies is crucial for addressing current limitations.

Further research can lead to personalized therapeutic approaches, tailoring interventions to individual patient needs, particularly for the unique characteristics of trisomy 21 in DS. Establishing explicit ethical guidelines and standardized safety protocols for these treatments is essential for broader acceptance and responsible clinical application. Increasing the number of large-scale controlled clinical trials could facilitate the transition of stem cell therapies from experimental interventions to standard treatment protocols. Despite existing challenges, stem cell therapy can potentially revolutionize treatment approaches for DS and AD. Continued research, interdisciplinary collaboration, and ethical discourse are required.

## Data Availability

The data supporting this study’s findings are available from the corresponding author upon reasonable request.

## Abbreviations

AD: Alzheimer’s disease
DS: Down syndrome
APP: Amyloid precursor protein
NSCs: Neural stem cells
MSCs: Mesenchymal stem cells
ESCs: Embryonic stem cells
iPSCs: Induced pluripotent stem cells
ASCs: Adult stem cells
HSCs: Hematopoietic stem cells
